# Agricultural Dust Exposures and Health and Safety Practices among Western Australian Wheatbelt Farmers during Harvest

**DOI:** 10.3390/ijerph16245009

**Published:** 2019-12-09

**Authors:** Krassi Rumchev, Suzanne Gilbey, Ryan Mead-Hunter, Linda Selvey, Kevin Netto, Ben Mullins

**Affiliations:** 1 School of Public Health, Curtin University, Perth 6148, Australia; sue.gilbey@curtin.edu.au (S.G.); r.mead-hunter@curtin.edu.au (R.M.-H.); l.selvey@uq.edu.au (L.S.); b.mullins@curtin.edu.au (B.M.); 2 School of Public Health, The University of Queensland, Herston 4006, Australia; 3 School of Physiotherapy and Exercise Science, Curtin University, Perth 6148, Australia; kevin.netto@curtin.edu.au

**Keywords:** particulate matter, agriculture, exposure, health and safety, farming, control measures, Australia

## Abstract

Background: Agricultural farmworkers are routinely exposed to high levels of airborne dust particles that have been linked to adverse health outcomes. Methods: This study measured personal and environmental exposures to dust particulates by farmworkers during harvesting activities. Farmers completed a workplace survey with regards to their health and safety awareness and practices and researchers observed general farm safety practices on selected farms using a checklist. Results: In this study, farmers were noted to commonly work extended hours and shifts during harvest due to rigid timing deadlines. Results showed that 40% of farmers were exposed to concentrations of inhalable particles greater than SafeWork Australia’s workplace exposure standards for grain dusts, assuming a 16 h working day over 5 shifts. Twenty-two percent were exposed to concentrations that were above the adjusted standard for 12 h shifts. Survey results showed that three-quarters of farm owners provided new workers with some type of induction related to farm safety, however this was mostly undertaken in an arbitrary manner. Despite noting that farming was a dusty occupation and reporting to use protective measures to reduce harmful dust exposures, no workers were observed to wear respiratory protection when working outside of the protection of a vehicle cabin. Conclusion: This study identified substantial gaps in health and safety knowledge among farm managers and workers, and improved education and training are highly recommended.

## 1. Introduction

Farming in Australia is considered a key industry with a fundamental role in contributing to the national and global economy [1]. Despite significant progress in farming technology, modern farming is still associated with many hazardous exposures, and is consistently identified as one of the most dangerous industries in which to work from a health and safety perspective [2,3,4,5]. In the Australian Work Health and Safety Strategy 2012–2022, agriculture was identified as a priority industry for further research to better understand hazardous exposures, along with the use and effectiveness of prevention actions and controls [6].

Whilst it is consistently acknowledged that the unique nature of farming involves co-exposures to multiple hazards [5,7,8,9], one of the main hazards is exposure to particulate air pollution generated during agricultural operations and processes [4]. In fact, agricultural workers are habitually exposed to high levels of particulates which are typically generated during field operations and consist of dusts originating from grain, pesticides, feed additives, fertilizers and biological aerosols from plant or animal matter [10,11,12,13,14].

Airborne dust particles or particulate matter (PM) may be characterized as dusts, fumes, smokes or mists, and is commonly classified according to its aerodynamic diameter (*d_a_*) with size fractions including coarse or PM_10_ with *d_a_* ≤ 10 μm, fine particles or PM_2.5_ with *d_a_* < 2.5 μm, and ultrafine particles (UFP) with *d_a_* < 0.1 μm.

Exposures to airborne dust particles derived from farming activities, including grain dusts, have consistently been associated with adverse respiratory health effects, including chronic cough, chronic phlegm, reduced lung function, shortness of breath and wheezing [13,14,15,16,17]. Grain dust is made up of complex mixtures of organic and inorganic materials mostly comprising of cellulose-based seed coatings and carbohydrate along with fungal and bacterial contaminants, endo- and mycotoxins, mites, insects and small quantities of crystalline silica [18]. Adverse health effects associated with grain dusts include a range of acute and chronic respiratory symptoms which manifests as reduced lung function. However, it is not known which agent or agents in grain dust are primarily responsible for the chronic respiratory health effects [18].

Whilst many of these health effects can be prevented or minimized by applying appropriate control measures [9], recent work by Cramer and colleagues [19] along with Schenker et al. [20] found that agricultural farm workers showed significant knowledge gaps regarding the identification of farming hazards including exposures to harmful dusts and chemicals, use of protective equipment and use of other control measures to reduce exposure.

Despite agriculture in Australia being recognized by SafeWork Australia [6] as a priority industry for further research, quantitative exposure assessment remains a poorly addressed area due to numerous complications. These can include large and diverse geographical areas along with rigid, and often short time frames for particular operations (e.g., harvesting, seeding, shearing). Furthermore, variability in degrees of dust generation and resultant exposure potential that exists is dependent on the type of agricultural operation being undertaken [11,16,21,22]. For example, during the seeding activities, the soil is typically damp and less likely to generate dust when compared to the harvesting period which is undertaken when the crop and soil are at their driest.

The primary objective of this study was to quantify personal and environmental exposures to grain dusts in Western Australian farm workers during harvest. Our secondary aim focused on understanding farm workers (including owners and managers) levels of health and safety knowledge related to identifying agricultural hazards, and the use of health and safety practices to reduce hazardous exposures.

## 2. Materials and Methods

This study is part of a larger project to investigate exposure to airborne dust particles, noise, vibration and chemicals in agricultural farm workers in Western Australia (WA). Farms were eligible for inclusion if they operated as grain or mixed grain-sheep farms; employed no more than five full-time equivalent farm workers; and acquired the majority of their income from agricultural activities.

**Figure 1:**
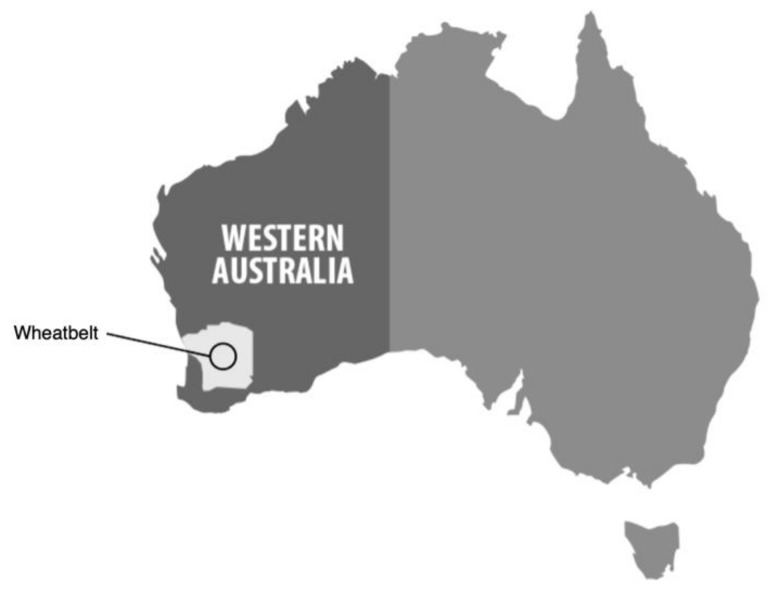
The Western Australian wheat-belt region.

A total of 29 farms meeting these criteria were recruited by telephone cold calling, through existing contacts of the WA Department of Agriculture and Curtin University, and by word-of-mouth from already recruited farms. Farms in the study were located in regions covering the entire WA wheat-belt (Figure 1) which is approximately 1600 km north to south and approximately 500 km east to west from the WA capital of Perth.

Data collection on participating farms was conducted during the 7-week grain harvesting period of October–December 2014 and included activities such as driving the harvester or chaser (the chaser is the vehicle that travels behind the harvester and where the grain is deposited in-field before being transferred to the grain delivery truck); grain delivery, field bin and auger use (the auger transfers the grain from the field bin to the grain delivery truck); truck driving, and various other miscellaneous harvesting-related tasks.

This study was approved by the Human Research Ethics Committee (HREC) of Curtin University with the Approval Number of SPH-81-2013.

### 2.1. Data Collection

Data was collected using a workplace survey, an on-site observation checklist, and dust monitoring. Researchers invited farmworkers and farm owners/managers to complete the survey if they were employed full time, over 18 years of age and able to provide written consent.

A workplace survey was jointly developed by Curtin University and Safe Work Australia based on existing, validated surveys and questionnaires [23,24,25] and included questions related to worker demographics, work experience and training, their knowledge of preventive measures applied on the farm to reduce risks, and worker use of preventative measures to reduce individual exposure to harmful dusts. Additional self-reported information gathered from farm owners and managers included farm characteristics (size, production activities, number of employees, use of contractors); provision of health and safety information and training; and preventative measures used to reduce dust exposures, such as provision of personal protective equipment (PPE). To ensure consistency, all surveys were administered face to face by researchers using an online survey tool (Qualtrics; Provo, Utah, US).

An on-site observation checklist based on existing work health and safety guidance materials for farming, and jointly developed by Curtin University and Safe Work Australia was also used to conduct independent observations on farms [23,24,25]. Due to limited access to farmers, researchers completed safety checklists where possible. Information collected during the farm observations included general safety, the availability of PPE, tractor safety, chemicals, and grain storage.

Quantitative measurements for personal and environmental exposures to agricultural dust were also undertaken as part of this study. Personal exposure to inhalable dust was monitored following the Australian Standard 3460–2009 (AS 3460–2009) using a plastic Institute of Occupational Medicine (IOM) sampler (SKC Inc., Eighty Four, PA, USA) connected to a sampling pump (SKC 210-1002MH, SKC Inc., Eighty Four, PA, USA). The IOM uses a reusable filter cassette containing a 25 mm polyvinyl chloride (PVC) filter (SKC Inc., Eighty Four, PA, USA) with a pore size of 5 µm. The IOM sampler was attached to the farmer’s clothes within their breathing zone and was operated at a flow rate of 2 L/min. Personal dust sampling was undertaken for a continuous 4 h period in accordance with AS 3460–2009.

In this study, dust exposures measured during harvesting operations could be considered as predominantly exposures to grain dusts and therefore the measured concentrations have been compared with the Safe Work Australia (SWA) workplace exposure standard for grain dust (oats, wheat and barley) of 4 mg/m^3^ [26].

Similar to other farming activities such as seeding, harvesting is weather dependent and where meteorological conditions are favourable, it is common for work shifts to be extended (beyond what is considered a conventional shift of 8 h per day) to between 12 and 16 h. Adjusted concentration values for extended shifts have been calculated using the Brief and Scala mathematical model [27]. This model serves as an estimation of likely exposures and adjusts the daily exposure standard (8 h) over 5 shifts per week (although it is acknowledged that workers can be working 7 days (shifts) per week during harvest).

In addition to personal dust sampling, environmental exposures to particulate air pollution was assessed inside the tractor’s cabin. Particulate matter in five size fractions including PM_1_, PM_2.5_, PM_4_, PM_10_, and total PM (TPM) was measured in real-time using a DustTrak monitor (8533, TSI, Shoreview, MN, USA). DustTrak operates based on a light scattering technique where the amount of scattered light is proportional to the volume concentration of the aerosol [28]. Dust concentrations were measured using a 37 mm PVC filter with a 5 μm pore size (SKC Inc., Eighty Four, PA, USA). DustTrak was operated at a sampling flow rate of 3.0 L/min and flow rate was calibrated prior to each sampling period using a mass flowmeter (4140, TSI Inc., Shoreview, MN, USA). The instrument was zero calibrated at each location prior to the commencement of operation, using the ‘zero cal’ function. The DustTrak was placed on clean cardboard in a position that would minimize disturbance of work processes and was programmed to log data at five-minute intervals. Environmental dust sampling was undertaken for a continuous 4 h period.

Consistent with a similar recent published study and described in Gilbey, et al. [29], the vehicle cabins have been considered as a micro-environment, and dust concentrations have been assessed against the adjusted PM concentrations for short-term exposures (1–4 h) which are based on the Australian 24 h National Environment Protection Measure (Ambient Air Quality) standards for PM_10_ and PM_2.5_. The adjusted 4 h concentrations are 0.075 mg/m^3^ and 0.060 mg/m^3^ for PM_10_ and PM_2.5_ respectively [30].

### 2.2. Data Analysis

Statistical analyses were conducted using the IBM SPSS statistical package (Version 21.0, IBM Corp, Armonk, NY, USA). Data collected by DustTrak was downloaded using the TrakPro software (TSI Inc., Shoreview, MN, USA) and was assessed for errors, validity, and reliability before further analyses. Continuous data was checked for normality and variables are presented as mean (± standard deviation; SD) and median (interquartile range; IQR). Categorical variables are described using frequencies and percentages.

Survey results were analysed in SPSS to provide summary statistics and also to perform linked comparisons with surveillance data and between survey and checklist data. Where comparisons were performed, the linkages were made on the basis of farm location rather than individual as identifying information about each individual was not available from questionnaires. Chi-square analysis and Fisher’s exact tests were used to determine if there were statistically significant differences between categorical variables. Non-significant results are not reported in the paper.

## 3. Results

### 3.1. Workplace Questionnaire

Twenty-nine mixed grain and livestock farms were visited during harvest and a total of 22 farm managers/owners, and 6 farm workers agreed to complete the questionnaire survey. Not all respondents answered every question. A response of ‘I don’t know’ or ‘not applicable’ was recorded however a lack of a response was treated as a missing value.

The majority of survey respondents were male (89%), aged between 25 and 68 years, with a median age of 53 years. Of the 28 survey respondents, four (14%) held an undergraduate level degree or higher, three (11%) had Tertiary and Further Education (TAFE), two (7%) had trade certificates however the majority (68%) did not have any formal education beyond high school.

Analyses of responses have generally been combined for farm owners/managers and farm workers for questions related to perceived dust sources, individual actions and use of control measures to reduce dust exposure and use of respiratory protective equipment (RPE). The survey for farm owners/managers included additional items on the provision of worker induction, sources of health and safety information and perceived causes of work-related illness and injury.

A total of 16 (73%) farm owners or managers reported employing farm workers and of these, 12 (75%) reported providing an induction to new workers, two (12.5%) reported doing this sometimes, and two (12.5%) never offered induction. Seven (32%) farm managers provided safety information to workers by walking around and talking to staff, five (23%) through informal discussions and two (9%) reported applying both. One (4%) farmer indicated that ‘work experience’ sufficed as induction. According to farm managers the most common source of information was gaining knowledge from other farmers, local newspapers, dedicated farming publications and from suppliers. Eight (36%) farm owners reported using a smartphone to obtain safety information via a mobile website.

In the questionnaire survey, farm owners were asked to select the three main causes of work-related injuries and illnesses on farms from a pre-defined list of 15 options. The most commonly reported causes included carelessness, long working hours, lack of education and training, unsafe work practices or procedures, and pressure or stress.

Farm owners and workers were also requested to report which activities they considered to be dustiest in farming. Most respondents (*n* = 24; 86%) reported that harvesting tasks undertaken outside an enclosed vehicle cabin were associated with high dust exposure, followed by field preparation or maintenance, cleaning or repairing machinery, shearing, working in stockyards and transferring grain (all: *n* = 23; 82%) (Table 1).

The most commonly reported preventive measure for minimizing dust exposure reported was keeping doors and windows closed when driving farm vehicles (74%). This was followed by regular maintenance, including maintenance and replacement of filters (58%), working upwind of dusty activities (53%), and providing washing facilities (32%) (Table 2).

The majority (89%) of farm owners/managers made disposable respirators available to workers—where known these were either P1 or both P1 and P2 (P1 retain approximately 80% of particles smaller than 2μm, P2 retain approximately 94% of particles smaller than 0.5μm) [31,32]. Six farms (32%) offered half-face or full-face respirators to workers. Only one farm provided a breathing apparatus with face masks or hoods and one farm did not offer any respiratory protection to farmworkers.

Farm workers (all respondents; *n =* 28) however reported to rarely use respiratory protection equipment (RPE), with the most commonly reported activity prompting the use of RPE (always or most of the time) being when transferring grain (39%) and when performing harvest tasks outside enclosed vehicles or tractors cabins (33%) (Table 3). Just over half of farm managers/owners (53%) reported to providing training to farmworkers in the correct use of RPE.

### 3.2. Farm Observations

Due to limited access to farms and facilities, independent observations on general work health and safety practices were conducted only on 19 farms (Table 4). Of the observed farms, 47% of the augers (a screw-type elevator that transports grain from the ground to the top of a grain bin or load truck) had undamaged guards fitted, and one-third of augers had winch cables without observable corrosion, wear or damage. None of the all-terrain vehicles (ATVs) were equipped with rollover protection devices (ROPS), and helmets were available for only one of the seven observed ATVs. Surveillance of grain storage practices also highlighted potential issues, with only 43% of silos being lockable to prevent unauthorized entry. Generally, the use of administrative controls such as warning signs on farms, or information relevant to hazard identification and risk assessment, were rarely observed by researchers. As an example, safety information on hazardous tasks such as ATV or chainsaw use, was available on 27% of the farms, whereas safety data sheets were readily accessible on 33%. Health and safety-related warning signs (e.g., chemical storage, explosives) that were secured and in good condition, were observed in 31% of farms.

### 3.3. Personal and Environmental Dust Exposure

Most personal exposures to inhalable grain dusts were below the SWA workplace exposure standard (WES) for grain dust of 4 mg/m^3^. One worker however, who was driving the chaser vehicle behind the harvester, was exposed to an elevated concentration of 5.35 mg/m^3^. Further results for all harvesting activities along with individual activities, are summarized at Table 5.

Farm observations and worker self-reports indicate that 12–16 h shifts were common during the harvesting period when timing deadlines and meteorological conditions dictate a tight harvesting schedule. For this reason, exposures based on an 8 h shift may not be meaningful. Using the Brief and Scala model to adjust exposure standards for extended working shifts (using a 5 day working week in the model), nine workers (22%) were exposed to inhalable particle concentrations that exceeded the adjusted exposure limit for a 12 h working day over five shifts, and another 16 workers (40%) were exposed to concentrations that would exceed the adjusted exposure limit for 16 h workdays over five shifts.

Environmental PM in five size fractions (PM_1_, PM_2.5_, PM_4_, PM_10_, TPM) was measured in real-time in the cabins of 24 vehicles during harvesting activities. Mean concentrations of PM_10_ for all harvesting activities were above the adjusted Western Australian Department of Health (WADOH) value of 0.075 mg/m^3^. Mean PM_2.5_ concentrations were all below the adjusted WADOH value of 0.060 mg/m^3^, apart from grain delivery which reported a mean of 0.16 mg/m^3^ (Table 6).

## 4. Discussion

In this study, we found that workers were exposed to high levels of grain and soil dust during harvest, particularly when taking into account the extended working hours commonly seen during this operation. Farmers reported to frequently work 12–16 h days over harvest, and when exposure standards were adjusted to reflect these extended shifts, it was estimated that 22% of workers were exposed to elevated inhalable dust concentrations during a 12 h workday (2 mg/m^3^), and 16 workers (40%) were exposed to higher levels than the adjusted standard of 1 mg/m^3^ if they worked a 16-h day. Whilst these adjusted values account for a 5 day working week, many workers reported to working 5+ days/shifts per week. It is therefore plausible that a greater number of farmers are exposed to agricultural dust concentrations exceeding the recommended values, which may contribute to adverse health effects [33,34,35,36].

Similar high PM_10_ concentrations were observed during the environmental monitoring with mean levels greater than the WADOH adjusted value of 0.075 mg/m^3^. The PM_2.5_ concentrations exceeded the WADOH adjusted value of 0.060 mg/m^3^ when workers were delivering grain.

The highest PM concentrations across all size fractions were consistently demonstrated when workers were undertaking tasks that involved out-of-cabin activities such as loading trucks from field bins and chaser bins (‘delivery’ and ‘using an auger’).

Despite the majority of farm owners/managers providing respiratory protection, the study findings showed that farm managers and workers rarely used RPE. Further, no evidence was provided to suggest that workers knew how to check for faults or correctly use respiratory protection. This is consistent with the findings of other studies [20,37] including a Californian study which reported that only 20% of farmers used RPE when undertaking dusty activities, although the more time spent undertaking ‘dusty’ activities, the less likely they were to use it [37].

The most-reported preventative measure used by farmworkers to minimize their dust exposure was to keep doors and windows closed when using farm vehicles. This is consistent with the in-cabin measurements of PM, however during the harvesting period, large portions of time are spent outside the cabin especially when undertaking ‘grain delivery’ tasks, with or without using an auger. Despite workers reporting to use protective measures to reduce dust exposure such as closing vehicle doors and windows, none of the workers were observed to incorporate any other control measures, such as the use of RPE to reduce or minimize dust exposure when working outside the protection of the vehicle cabin.

Whilst farmers and workers seemed to infer a level of health and safety knowledge, the study results suggest that farmers and farmworkers are not fully informed of the potential hazards and associated risks in relation to harmful exposures. This concept is however not new as the findings of a recent study by Cecchini, Bedini, Mosetti, Marino and Stasi [4] indicated that farmers may not appreciate the risk of exposure to hazards in their working environment and that workers do not generally consider their work to be dangerous. In our study, 32% farmers provided safety training by walking around the work area and another 23% through informal discussions.

This study acknowledges that farm work involves a diverse range of tasks, crops and meteorological and climatic conditions making it difficult to apply generalizations regarding harmful dust exposures across other geographically different agricultural environments. It should be recognized that this study was undertaken in the WA wheat-belt along with its unique climatic conditions, and the observed exposures may differ to those in other Australian agricultural regions. It is, however, considered that the findings of this study might have relevance to farmers across Australia and other countries engaged in similar processes and types of operation or farming under similar crop, climatic and geographical conditions. Similar caution regarding study generalizability has been reported in other agricultural studies [38,39], although colleagues in New Zealand [7,40] indicate that estimations of personal dust concentrations may be possible for exposures where agricultural operations are carried out under similar conditions.

One of the strengths of this study included that personal exposure samples were achieved despite workers reporting the hot, dry conditions during harvest time made the process somewhat uncomfortable (which is also the reason many of the workers reported for not wearing RPE). For this reason, only 4 h, albeit continuous, of monitoring was achievable for each participant. We acknowledge this as a limitation of the study; however, consider that the 4 h period is likely to be a reasonable representation of exposures given most workers reported task homogeneity during their monitoring period.

This study also acknowledges limitations in relation to variable weather conditions and diversely located farms and paddocks resulting in frequent, short-notice changes in farm work and locations. Additionally, activities in the annual farming cycle such as harvesting are subject to rigid time frames. In the case of this study, an intense 7 week harvesting period was planned to coincide with the crop being at its driest. Whilst most workers reported task homogeneity for the entire 4 h sampling period, in reality, the sampling period could involve a combination of tasks guided by the previously noted difficulties. Some challenges were presented by the reticence of the study population to participate however this, and previously noted limitations, are not unique and have been reported as challenges in other similar studies [11,38].

Although these combined factors resulted in small sample size, the farms involved in the study were randomly selected from a large representative region of the Western Australian wheat-belt. This however limited the ability to investigate in greater detail any observed variability in survey responses, and exposure measurements.

These limitations are common with other similar international farming exposure studies including studies conducted in California agriculture environment [11,39,40].

A different study design may provide a larger dataset, which could better identify significant associations between tasks, exposures and determinants.

## 5. Conclusions

This research found that farmers and farmworkers were exposed to high levels of airborne dust particles. We also observed significant gaps in health and safety knowledge amongst farmers and farm workers and future studies that focus on more comprehensive education and training to farmers and workers is highly recommended. This may involve improved induction and ongoing health and safety updates which aim to increase hazard identification capabilities. Education and training may encourage the adopting of work processes and behaviours among farm workers that will not only limit hazardous exposures but might also translate to the identification of other hazardous elements and situations within the agricultural environment, and subsequent remediation to mitigate adverse outcomes. Noting that the majority of farm owners (36%) reverted to using their smartphones and mobile websites to obtain safety information, future training and education should include digital-based resources (e.g., apps, online induction). Online education and training may also provide significantly further benefits by enabling the identification and tracking of potential health and safety knowledge gaps existing amongst workers.

This is a position supported by recent research with Cramer, Wendl, Sayles, Duysen and Achutan [19] suggesting that the attitude and behaviour of farmers towards health and safety practices may change by applying appropriate education and training, which then can lead to improved agricultural safety and health.

## Figures and Tables

**Table 1:** Activities reported to be dusty in farming (*n =* 28).

Activity.	Yes (%)	No	Don’t Know
Harvesting Outside Enclosed Cabin	24 (86)	0	1
Stubble or Windrow Burning	19 (68)	1	0
Field Preparation or Maintenance	23 (82)	0	1
Cleaning or Repairing Machinery	23 (82)	0	1
Using or Moving Farm Vehicles or Equipment	20 (71)	2	2
Shearing	23 (82)	2	0
Working in Stockyards	23 (82)	0	0
Transferring Grain	23 (82)	1	0

**Table 2:** Reported methods to reduce exposure to dust during typical farming tasks (*n* = 19).

Task	*n* (%)
Use machinery or equipment that collects or extracts dusts	0 (0)
Extracted air is vented outdoors away from workers, doors, and windows	3 (16)
Regular maintenance, including replacing filters	11 (58)
Use ventilation systems in indoor work areas	2 (11)
Keep doors and windows closed when using farm vehicles with enclosed cabins	14 (74)
Ensure farm workers are upwind of dusty activities	10 (53)
Restrict access to those areas where dusty tasks are done	5 (26)
Rearrange work areas so dusty tasks are carried out away from other workers	5 (26)
Limit the amount of time you or your farm workers carry out dusty tasks, i.e., through job swapping	4 (21)
Use warning signs to remind farm workers to wear respiratory protective equipment	0 (0)
Use vacuum cleaners to remove dusts	0 (0)
Wet down dusts before sweeping if vacuum cleaners can’t be used	3 (16)
Provide overalls or disposable overalls so dusts aren’t taken into rest or accommodation areas	0 (0)
Provide washing facilities	6 (32)

**Table 3:** Reported use of respiratory protection (all respondents, *n* = 28).

Activity	Always or Mostly (%)	Sometimes	Never	No Response or Don’t Do This Task
Harvesting outside enclosed cabin	7 (33)	3	11	7
Stubble or windrow burning	3 (15)	2	14	9
Field preparation or maintenance	1 (6)	2	14	11
Cleaning or repairing machinery	6 (30)	6	8	8
Using or moving farm vehicles or equipment	0 (0)	2	15	11
Shearing	1 (7)	1	12	14
Working in stockyards	1 (6)	1	13	13
Transferring grain	7 (39)	2	9	10

**Table 4:** On-site work health and safety observations (researcher checklist).

Checklist Items	*n*	Observed (%)
**General Safety**		
Safety information like risk assessments or Safety Data Sheets (SDS) is available for all hazardous jobs on the farm	15	4 (27)
Emergency procedures like evacuation plans and emergency contacts are readily identifiable	11	5 (46)
Procedure, plant and equipment manuals are current and available in the area where tasks are carried out	16	10 (63)
Areas where hazardous tasks like hot work or chemical mixing are carried out are separated from general work areas	11	7 (64)
Warning and safety signs are in good condition and secured	13	4 (31)
Signs display personal protective equipment (PPE) requirements where required in the area where tasks are carried out	12	1 (8)
Firefighting equipment such as extinguishers and blankets are easily accessible and maintained	18	15 (83)
Eye wash is easily accessible	14	9 (64)
First aid kit is easily accessible and fully stocked	17	10 (59)
Residual Current Device (RCD) is fitted to the workshop electrical system	13	2 (15)
Work area can be opened from the inside without a key	10	10 (100)
General workplace organisation		
Adequate storage area provided for work items and equipment	18	15 (83)
**Harvesting—Augers**		
Undamaged guards fitted so that fingers, feet and clothing cannot be caught	15	7 (47)
Winch cable is free of corrosion, wear, or damage	12	4 (33)
Warning signs placed near power lines where portable augers/elevators are located or generally used	11	1 (9)
Appropriate PPE like ear muffs and dust masks are available when using augers	14	10 (71)
**Harvesting—Combine Harvesters**		
Harvesters have cabins	6	6 (100)
Cabins are air-conditioned	6	5 (83)
Air-conditioners have dust filters	5	5 (100)
Harvesters are equipped with fire extinguishers that are secured and maintained	11	10 (91)
Ladders, steps or safe access platforms are in good repair and free of mud and grease	12	11 (92)
Appropriate PPE like ear muffs and dust masks are available during harvesting operations	11	7 (64)
**Tractor Safety**		
Tractors have cabins	11	11 (100)
Cabins are air-conditioned	11	9 (82)
Air-conditioners have dust filters	10	9 (90)
All tractors are fitted with Roll Over Protection Structure (ROPS) that meet Australian Standards	16	16 (100)
Guards and shields including the master Power Take-Off (PTO) shield are fitted and securely fastened	16	13 (81)
Seat safety switches are connected and functional to prevent the tractor from being ‘jumped started’ from the ground	14	11 (79)
All headlights, flashers, and brake lights are working correctly, clean, and visible to other drivers or workers	17	17 (100)
Steps, ladders, safe access platforms and cab areas are free from mud, dirt, oil, or any other combustible object or fluid	16	14 (88)
Firefighting equipment is securely fastened inside the cab or operator’s station	15	14 (93)
A first aid kit securely fastened inside the cab or operator’s station and is fully stocked	14	5 (36)
Cab windows and mirrors are undamaged, clean and provide good visibility	17	17 (100)
**All-Terrain Vehicle (ATV) Safety**		
ATVs are equipped with ROPS	7	0 (0)
Helmets are available when ATVs are used	7	1 (14)

**Table 5:** Personal inhalable particle concentrations for harvesting activities (PMi) (*n* = 40).

Sub-Task	*n*	Concentration (mg/m^3^)	
		Mean ± SD	Min–Max
All harvesting	40	1.20 ± 1.09	0.15–5.35
Header (harvester)	18	1.09 ± 0.75	0.23–2.54
Chaser	11	1.21 ± 1.46	0.15–5.35
Grain delivery ^a^	9	1.46 ± 1.33	0.30–3.64
Using an auger ^a^	2	1.04 ± 0.35	0.80–1.29

^a^ Using an auger always involved driving a grain truck, however using an auger was noted to form a significant part of the task. ‘Grain delivery’ did not involve use of an auger.

**Table 6:** Real-time concentrations for all harvesting and individual harvesting tasks (4 h) (*n =* 24).

9		Concentration (mg/m^3^)	
Task	PM Size Fraction	Mean ± SD	Min–Max
All harvesting (*n =* 24)			
	TPM	0.55 ± 0.80	0.00–3.84
	PM_10_	**0.15 ± 0.22**	0.00–1.11
	PM_4_	0.05 ± 0.10	0.00–0.51
	PM_2.5_	0.05 ± 0.09	0.00–0.46
	PM_1_	0.04 ± 0.09	0.00–0.44
Harvester (*n =* 12)			
	TPM	0.30 ± 0.46	0.00–1.66
	PM_10_	**0.07 ± 0.07**	0.00–0.24
	PM_4_	0.02 ± 0.02	0.00–0.09
	PM_2.5_	0.02 ± 0.02	0.00–0.09
	PM_1_	0.02 ± 0.02	0.00–0.08
Chaser (*n =* 5)			
	TPM	0.45 ± 0.57	0.00–1.30
	PM_10_	**0.10 ± 0.12**	0.00–0.32
	PM_4_	0.02 ± 0.01	0.00–0.03
	PM_2.5_	0.02 ± 0.01	0.00–0.03
	PM_1_	0.02 ± 0.01	0.00–0.03
Grain delivery (*n =* 6)			
	TPM	1.45 ± 1.32	0.00–3.84
	PM_10_	**0.47 ± 0.35**	0.00–1.11
	PM_4_	0.17 ± 0.20	0.00–0.51
	PM_2.5_	**0.16 ± 0.18**	0.00–0.46
	PM_1_	0.15 ± 0.17	0.00–0.44
Using an auger (*n =* 1 *)			
	TPM	0.44	
	PM_10_	**0.08**	
	PM_4_	0.02	
	PM_2.5_	0.01	
	PM_1_	0.01	

* Sample size is too small to calculate SD; concentrations in **bold** signify they exceed WADOH adjusted values; SD: standard deviation; TPM: total particulate matter.
